# Factors Associated with Emergency Department Revisit and Urologic Intervention Following Discharge of Renal Colic Patients: A Retrospective Cohort Study from a Military Hospital in the Kingdom of Bahrain

**DOI:** 10.7759/cureus.117168

**Published:** 2026-09-29

**Authors:** Fatima H Almuhanadi, Rashed Buhaza, Ali S Buallay, Ahmed Alabsi, Hamad Alnajem, Hessa AlBuainain

**Affiliations:** 1 Emergency Medicine, Bahrain Defence Force Hospital, Riffa, BHR; 2 General Practice, Bahrain Defence Force Hospital, Riffa, BHR

**Keywords:** bahrain, emergency department revisit, renal colic, risk stratification, ureterolithiasis, urologic intervention

## Abstract

Background

Renal colic is one of the most frequent urologic presentations to the emergency department (ED). While most patients can be safely discharged with symptomatic management, a subset return to the ED or require urologic intervention within 14 days. Identifying factors associated with these outcomes is essential for informed disposition planning.

Objectives

The objectives of the study are to determine 14-day ED revisit and urologic intervention rates among discharged renal colic patients and to identify independent clinical and imaging predictors of each outcome.

Methods

A retrospective cohort study of patients aged ≥14 years discharged with renal colic from the Royal Medical Services (RMS) Military Hospital ED, Bahrain, in 2025. Demographic, clinical, laboratory, and CT imaging data were extracted. Univariate and multivariable logistic regression analyses were performed.

Results

In this study, 609 patients were included (mean age 42.5±12.7 years; 71.4% men). The 14-day ED revisit rate was 18.4% (n=112) and the urologic intervention rate was 14.1% (n=86). Independent predictors of ED revisit were male sex (adjusted odds ratio (aOR) 1.79; 95% confidence interval (CI) 1.06-3.01), previous stone history (aOR 1.88; 95% CI 1.22-2.89), and opioid use at index visit (aOR 2.11; 95% CI 1.31-3.40). Stone size ≥5-7 mm and presence of obstruction independently predicted urologic intervention. Pain severity was not a predictor of either outcome.

Conclusion

Opioid use, prior stone history, and male sex predict ED revisit; stone size and obstruction predict urologic intervention. These findings support risk stratification and discharge planning for renal colic patients.

## Introduction

Renal colic, resulting from urinary tract calculi, is among the most painful and frequently encountered conditions in emergency medicine, accounting for approximately 1%-2% of all emergency department (ED) presentations globally [1]. The condition imposes a substantial burden on emergency services through high rates of diagnostic imaging, analgesic consumption, and short-term return visits. In the Gulf Cooperation Council (GCC) region, including Bahrain, urolithiasis is particularly prevalent owing to climatic, dietary, and genetic factors, with epidemiological studies suggesting lifetime stone recurrence rates exceeding 50% in affected individuals [2,3].

Emergency physicians must routinely make discharge decisions under time pressure and clinical uncertainty. The majority of patients with uncomplicated renal colic can be managed with analgesia and safely discharged, with expectant management of spontaneous stone passage [4,5]. However, a clinically important subset will return to the ED within days of discharge - either due to inadequate pain control, stone impaction, complications such as infection or acute kidney injury, or the need for definitive urologic intervention. These revisits contribute to ED crowding, increased healthcare costs, and patient dissatisfaction [6].

ED revisit rates following discharge for renal colic have been reported in the range of 10%-30% in international literature, while urologic intervention rates within 30-60 days vary from 10% to 25% depending on stone characteristics and follow-up practices [7,8]. Despite this, most published studies focus on procedural outcomes rather than ED revisit as an independent quality endpoint. ED revisit is a recognized quality metric in emergency medicine, reflecting both clinical progression and the adequacy of initial discharge planning [6].

A persistent gap exists regarding contemporary, ED-specific evidence from Middle Eastern settings, where stone disease patterns, analgesic preferences, and institutional disposition practices may differ from Western cohorts [7]. This study addresses that gap by evaluating predictors of ED revisit and urologic intervention among patients discharged from the Royal Medical Services (RMS) Military Hospital ED in the Kingdom of Bahrain.

Background and rationale

Symptomatic nephrolithiasis and ureterolithiasis represent a globally increasing health burden, with lifetime prevalence of 10%-15% in developed nations and likely higher in arid climates such as Bahrain [2]. Acute renal colic results from obstruction or irritation of the urinary tract by calculi, producing severe colicky flank or abdominal pain often associated with nausea, vomiting, and urinary symptoms. ED management centers on analgesia - with nonsteroidal anti-inflammatory drugs (NSAIDs), paracetamol, and opioids - alongside imaging and disposition planning [4,5].

Studies comparing direct ED discharge with observation unit management have demonstrated similar short-term revisit and intervention rates, supporting the safety of discharge in appropriately selected patients [6]. Large retrospective cohort studies have established that stone size and proximal ureteral location are among the most robust predictors of urologic intervention, with stones ≥6 mm and proximal stones being substantially less likely to pass spontaneously [7,8].

Pain severity at initial presentation has not been shown to correlate reliably with stone burden or downstream outcomes [9]. Analgesic requirement, particularly the need for opioid analgesia, may be a more clinically relevant marker [4]. CT imaging remains the gold standard for stone characterization [10]; however, CT-derived findings are only available in patients who undergo imaging, limiting their applicability to the full ED population.

This study treats both ED revisit and urologic intervention as co-primary outcomes within a 14-day follow-up window, using a contemporary dataset from the RMS Military Hospital ED. The findings are intended to support evidence-based risk stratification and institutional follow-up pathway development for renal colic patients.

Study objectives

Primary Aim

The primary aim is to identify patient and stone characteristics independently associated with (1) emergency department revisit and (2) urologic intervention within 14 days of discharge among patients aged ≥14 years presenting with renal colic to the RMS Military Hospital ED, Bahrain.

Specific Objectives

The specific objectives were: to determine the 14-day ED revisit rate among patients discharged with renal colic; to determine the 14-day urologic intervention rate following ED discharge; to identify pre-imaging clinical predictors - including age, sex, pain severity, prior stone history, associated symptoms, and analgesic use - of both outcomes; to identify post-imaging stone-related predictors, including stone size, location, and degree of obstruction; and to develop multivariable models identifying independent predictors of each outcome after adjusting for confounders.

## Materials and methods

Methodology

Study Design

This was a retrospective cohort study using electronically extracted data from the Emergency Department of the Royal Medical Services (RMS) Military Hospital, Kingdom of Bahrain. The study adhered to the STROBE (Strengthening the Reporting of Observational Studies in Epidemiology) reporting guidelines for observational research [11].

Setting

The RMS Military Hospital ED serves both a military and civilian population in the Kingdom of Bahrain, with 24-hour CT imaging capability and on-call urology services. The study period covered the calendar year 2025.

Study Population

Patients aged ≥14 years - which is consistent with our institution's established clinical definition of the adult ED population - presenting to the ED with a primary discharge diagnosis of renal colic or ureterolithiasis (International Classification of Diseases, 10th revision (ICD-10): N20-N23) were eligible for inclusion, provided they were discharged from the ED at the index visit and had available 14-day follow-up data within the hospital information system. Patients were excluded if they were admitted to hospital during the index visit, had an alternative diagnosis identified, carried a diagnosis of end-stage renal disease (defined by chronically elevated creatinine or dialysis dependence), had undergone a urologic intervention within the preceding month (e.g., Double-J (DJ) stent insertion, ureteroscopy, or extracorporeal shock wave lithotripsy (ESWL)), or had incomplete records precluding outcome ascertainment.

Sample Size

A target sample size of 500 patients was pre-specified in the study protocol, based on an anticipated ED revisit rate of approximately 15%-20% [6,7] and a minimum detectable odds ratio of 1.8 for key predictors with 80% power at a two-sided significance level of 0.05. Following data extraction and application of eligibility criteria, a final cohort of 609 patients was available for analysis.

Data Collection

Data were extracted from the hospital electronic medical records (EMR) system using a standardized data extraction form. CT imaging was performed at the discretion of the treating emergency physician according to institutional practice and clinical presentation; imaging was not mandated by the study protocol. Extracted variables included demographics (age, sex); clinical presentation data encompassing triage pain severity score (numeric rating scale 0-10), associated symptoms (nausea/vomiting, urinary symptoms, both, or none), and previous history of renal calculi; ED management data including analgesics administered (paracetamol, NSAID, opioid) and renal function test (RFT) results; imaging data including CT performance (yes/no), stone size category (<5 mm, 5-7 mm, >7 mm), stone location (renal, proximal ureter, mid ureter, distal ureter, bladder), and degree of obstruction (non-obstructing, mild, moderate, severe); disposition (discharged, referred to outpatient clinic, or referred to on-call urology); and both primary outcome variables.

Outcome Definitions

The primary outcomes are the following:

(1) ED revisit: Any unplanned return to the RMS Military Hospital ED within 14 days of the index discharge with a presenting complaint related to renal colic or urinary calculi.

(2) Urologic intervention: Performance of any urologic procedure within 14 days of ED discharge, including ureteroscopy, extracorporeal shock wave lithotripsy (ESWL), percutaneous nephrolithotomy (PCNL), or ureteric stent (DJ stent) insertion.

A 14-day follow-up window was selected to capture early failure of conservative management, including recurrent uncontrolled symptoms, repeated emergency department utilization, and the need for expedited urologic intervention. Patients who experienced both outcomes were classified in both outcome groups for their respective analyses.

Statistical Analysis

All data were extracted, cleaned, and standardized prior to analysis. Free-text categorical entries were standardized by two independent reviewers. Statistical analysis was performed using Python 3.x (pandas, scipy, numpy libraries; Python Software Foundation, Fredericksburg, VA). Continuous variables are presented as mean±SD or median (interquartile range (IQR)) depending on distribution. Categorical variables are presented as frequencies and proportions. Univariate associations between candidate predictors and each outcome were assessed using Fisher's exact test for 2×2 categorical comparisons, Pearson chi-square for larger contingency tables, and Mann-Whitney U test for continuous variables. Test statistics (U, χ²) and degrees of freedom are reported alongside p-values. Variables with p<0.10 on univariate analysis were entered into multivariable binary logistic regression models for each outcome. Model fit was assessed using the Nagelkerke R² and the overall model chi-square. Results are reported as adjusted odds ratios (aOR) with 95% confidence intervals. Statistical significance was set at p<0.05. Missing data were handled by complete-case analysis; the urologic intervention model was restricted to CT-imaged patients (n=175) as stone characteristics were unavailable for the remainder.

Ethical Considerations

The study was conducted in accordance with the Declaration of Helsinki. Ethical approval was obtained from the Royal Medical Services (RMS) Institutional Review Board prior to data extraction. Patient data were de-identified prior to analysis. Individual patient consent was waived given the retrospective observational design.

## Results

Study cohort and baseline characteristics

A total of 609 patients met the inclusion criteria and were enrolled in the study. The cohort was predominantly made up of men (435 patients, 71.4%), with a mean age of 42.5±12.7 years (median 41 years, IQR 33-51). The median triage pain severity score was 5 (IQR 4-6). Nearly half of all patients (295, 48.4%) had a documented prior history of renal calculi. The most common associated symptom was urinary symptoms alone (232, 38.1%), followed by combined urinary and nausea/vomiting symptoms (167, 27.4%). Renal function tests were performed in 539 patients (88.5%), of whom 129 (23.9%) had an abnormal result. CT imaging was performed in 219 patients (36.0%), with a stone identified in 198 patients (90.4% of CT scans). Of 198 patients with stone identified on CT, 175 had complete data for multivariable analysis; 23 patients were excluded because of missing data required for the model. Among stones identified, the majority were small (<5 mm; 143, 72.2%), with 48 (24.2%) measuring 5-7 mm and seven (3.5%) exceeding 7 mm. The most common stone location was the distal ureter (116, 56.9%). Opioid analgesia was administered in 358 patients (58.8%). Analgesic use is illustrated in Figure 1. Baseline characteristics are presented in Table 1.

**Figure 1:**
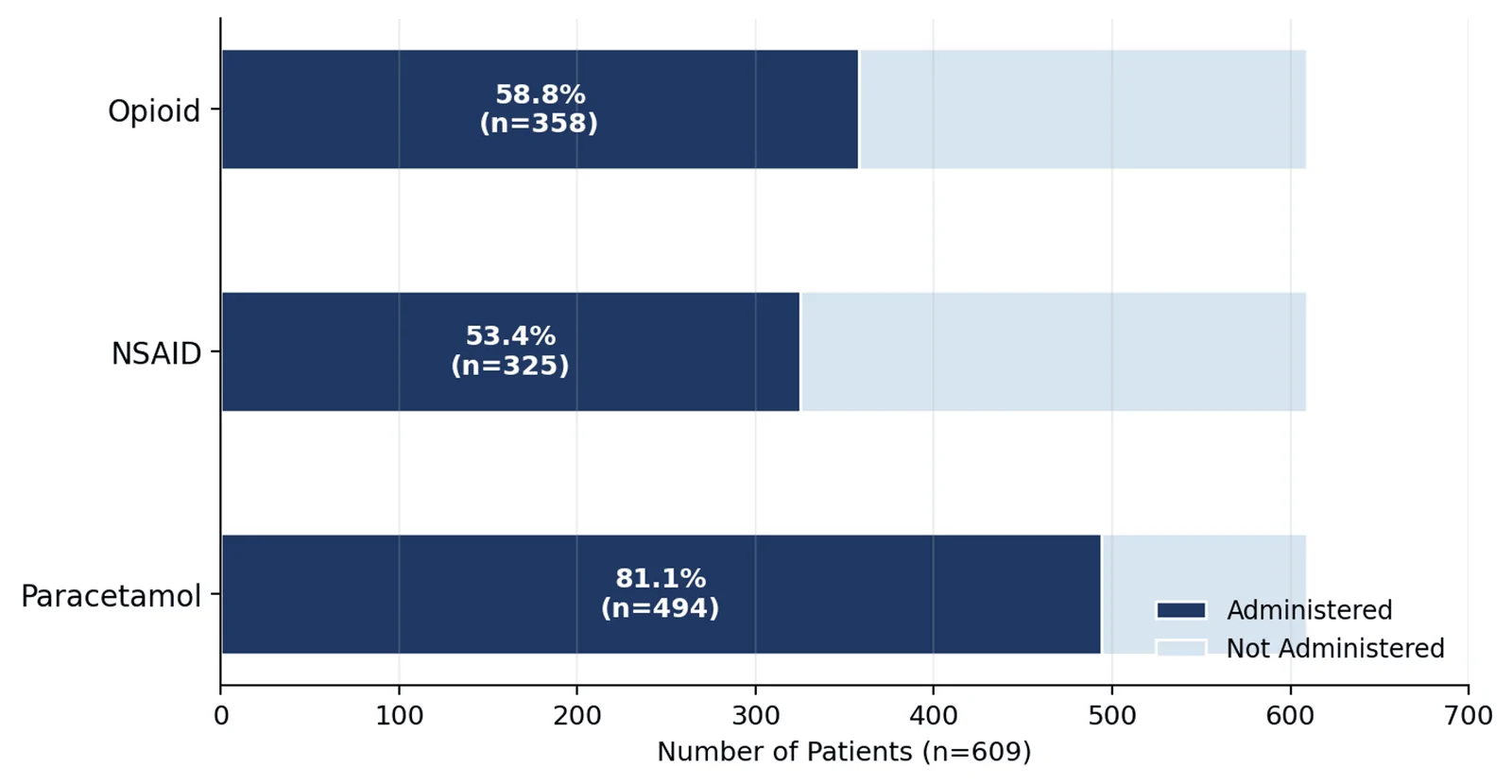
Analgesic use at the index emergency department visit (n=609). Bars show administered (navy) and not administered (light blue) proportions. Percentage and absolute count are displayed for each agent. NSAID: Non-steroidal anti-inflammatory drugs.

**Table 1:** Baseline Demographic and Clinical Characteristics (n=609) Proportions for associated symptoms and stone characteristics are calculated from patients with available data. CT: computed tomography; RFT: renal function tests; IQR: interquartile range; SD: standard deviation.

Characteristic	n (%) or Median (IQR)
Demographics	
Male sex	435 (71.4%)
Age, years - median (IQR)	41 [33–51]
Age, years - mean±SD	42.5 ± 12.7
Clinical presentation	
Pain severity score - median (IQR)	5 [4–6]
Previous history of renal stones	295 (48.4%)
Associated symptoms: Urinary symptoms only	232 (38.1%)
Associated symptoms: Nausea/Vomiting only	98 (16.1%)
Associated symptoms: Both	167 (27.4%)
Associated symptoms: None	101 (16.6%)
Analgesic use	
Paracetamol	494 (81.1%)
NSAID	325 (53.4%)
Opioid	358 (58.8%)
Investigations	
RFT performed	539 (88.5%)
RFT abnormal (of those tested)	129 (23.9%)
CT imaging performed	219 (36.0%)
Stone identified on CT	198 (90.4% of CT scans)
Stone characteristics (n=198 with stone on CT)	
Size <5 mm	143 (72.2%)
Size 5-7 mm	48 (24.2%)
Size >7 mm	7 (3.5%)
Location: Distal Ureter	116 (56.9%)
Location: Proximal Ureter	34 (16.7%)
Location: Mid Ureter	16 (7.8%)
Location: Renal	31 (15.2%)
Obstruction: Non-obstructing	38 (19.7%)
Obstruction: Mild	115 (59.6%)
Obstruction: Moderate	36 (18.7%)
Obstruction: Severe	4 (2.1%)
Disposition	
Discharged	181 (29.8%)
Referred to Outpatient Clinic	279 (45.9%)
Referred to On-Call Urology	144 (23.7%)

Primary outcomes

Within 14 days of discharge, 112 patients (18.4%) revisited the ED and 86 patients (14.1%) underwent urologic intervention. A total of 20 patients (3.3%) experienced both outcomes. The majority of the cohort (430 patients, 70.6%) had no adverse outcome within the follow-up window. 

Univariate analysis: ED revisit

On univariate analysis, three variables were significantly associated with 14-day ED revisit: male sex (81.3% vs 69.2%; χ²=5.97, df=1, p=0.015), previous history of renal stones (62.5% vs 45.4%; χ²=9.95, df=1, p=0.002), and opioid use at the index visit (73.2% vs 55.6%; χ²=10.37, df=1, p=0.001). Age (U=26,182, p=0.342), pain severity score (U=27,880, p=0.868), NSAID use (χ²=0.10, p=0.748), CT imaging (χ²=0.42, p=0.519), RFT result (χ²=2.22, p=0.136), associated symptoms (χ²=0.16, df=3, p=0.984), and stone characteristics (stone size: χ²=2.23, p=0.328; location: χ²=4.32, p=0.364; obstruction: χ²=1.67, p=0.643) were not significantly associated with revisit. Results are presented in Table 2 and Figure 2.

**Table 2:** Univariate Analysis: ED Revisit within 14 Days (n=609) *p<0.05. Fisher exact test or chi-square (χ²) for categorical variables; Mann-Whitney U for continuous variables. NSAID: Non-steroidal anti-inflammatory drug; RFT: renal function test.

Variable	No Revisit (n=497)	Revisit (n=112)	Test Statistic / p-value
Age, median (IQR)	42 (34-52)	40 (32-49)	U=26,182; p=0.342
Male sex	343 (69.2%)	91 (81.3%)	χ²=5.97, df=1; p=0.015*
Previous history of stones	225 (45.4%)	70 (62.5%)	χ²=9.95, df=1; p=0.002*
Pain severity, median (IQR)	5 (4-6)	5 (4-6)	U=27,880; p=0.868
Opioid use	276 (55.6%)	82 (73.2%)	χ²=10.37, df=1; p=0.001*
NSAID use	263 (53.0%)	62 (55.4%)	χ²=0.10, df=1; p=0.748
CT imaging performed	175 (35.3%)	44 (39.3%)	χ²=0.42, df=1; p=0.519
RFT abnormal	111 (27.1%)	18 (17.1%)	χ²=2.22, df=1; p=0.136
Associated symptoms	-	-	χ²=0.16, df=3; p=0.984
Stone size (CT+subgroup)	-	-	χ²=2.23, df=2; p=0.328
Stone location (CT+subgroup)	-	-	χ²=4.32, df=4; p=0.364
Obstruction degree (CT+subgroup)	-	-	χ²=1.67, df=3; p=0.643

**Figure 2:**
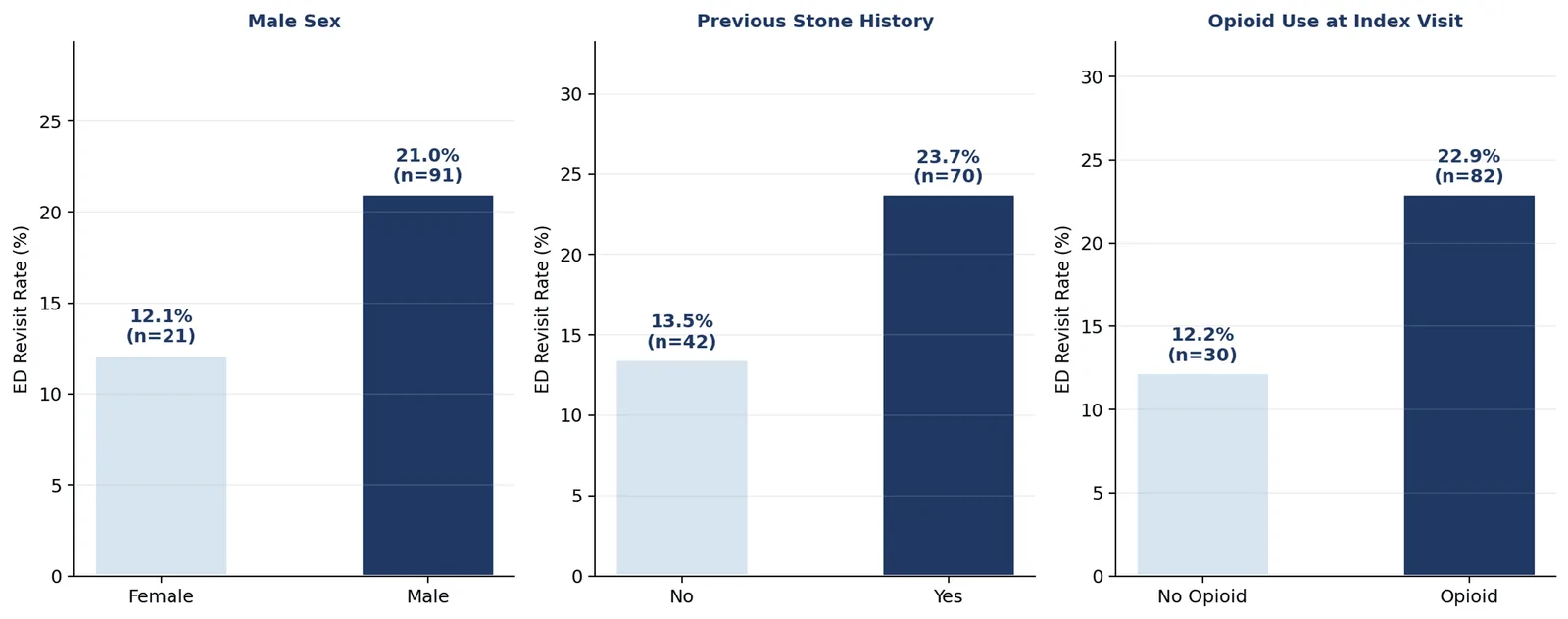
Fourteen-day ED revisit rate by independent clinical predictors. Each bar displays the revisit rate (%) with absolute count (n) of revisit patients. The three predictors shown were each statistically significant on both univariate and multivariable analysis.

Univariate analysis: urologic intervention

Multiple factors were significantly associated with urologic intervention on univariate analysis: older age (median 47 vs 41 years; U=25,620, p=0.035), previous stone history (58.1% vs 46.9%; χ²=3.67, df=1, p=0.055), opioid use (80.2% vs 55.4%; χ²=17.25, df=1, p<0.001), CT imaging performed (77.9% vs 29.1%; χ²=73.42, df=1, p<0.001), abnormal RFT (44.2% vs 20.1%; χ²=24.99, df=1, p<0.001), stone size [7,8] (χ²=17.02, df=2, p<0.001), stone location (χ²=22.28, df=4, p<0.001), and degree of obstruction (χ²=17.01, df=3, p=0.001). Male sex (χ²<0.01, p=0.977) and pain severity score (U=21,966, p=0.812) were not significantly associated [9]. The results are presented in Table 3 and Figures 3, 4.

**Table 3:** Univariate Analysis: Urologic Intervention within 14 Days (n=609) *p<0.05. Fisher exact test or chi-square (χ²) for categorical variables; Mann-Whitney U for continuous variables.

Variable	No Intervention (n=523)	Intervention (n=86)	Test Statistic / p-value
Age, median (IQR)	41 (33-50)	47 (35-54)	U=25,620; p=0.035*
Male sex	372 (71.3%)	62 (72.1%)	χ²<0.01, df=1; p=0.977
Previous history of stones	245 (46.9%)	50 (58.1%)	χ²=3.67, df=1; p=0.055
Pain severity, median (IQR)	5 (4-6)	5 (4-6)	U=21,966; p=0.812
Opioid use	289 (55.4%)	69 (80.2%)	χ²=17.25, df=1; p<0.001*
CT imaging performed	152 (29.1%)	67 (77.9%)	χ²=73.42, df=1; p<0.001*
RFT abnormal	91 (20.1%)	38 (44.2%)	χ²=24.99, df=1; p<0.001*
Associated symptoms	-	-	χ²=7.80, df=3; p=0.050
Stone size (CT+)	—	—	χ²=17.02, df=2; p<0.001*
<5 mm	107 (82.3%)	36 (52.9%)	
5-7 mm	26 (20.0%)	22 (32.4%)	
>7 mm	1 (0.8%)	6 (8.8%)	
Stone location (CT+)	-	-	χ²=22.28, df=4; p<0.001*
Proximal Ureter	14 (10.8%)	20 (29.4%)	
Mid Ureter	9 (6.9%)	7 (10.3%)	
Distal Ureter	79 (60.8%)	37 (54.4%)	
Renal	28 (21.5%)	3 (4.4%)	
Obstruction degree (CT+)	-	-	χ²=17.01, df=3; p=0.001*
Non-obstructing	36 (27.7%)	2 (2.9%)	
Mild	75 (57.7%)	40 (58.8%)	
Moderate	19 (14.6%)	17 (25.0%)	
Severe	3 (2.3%)	1 (1.5%)	

**Figure 3:**
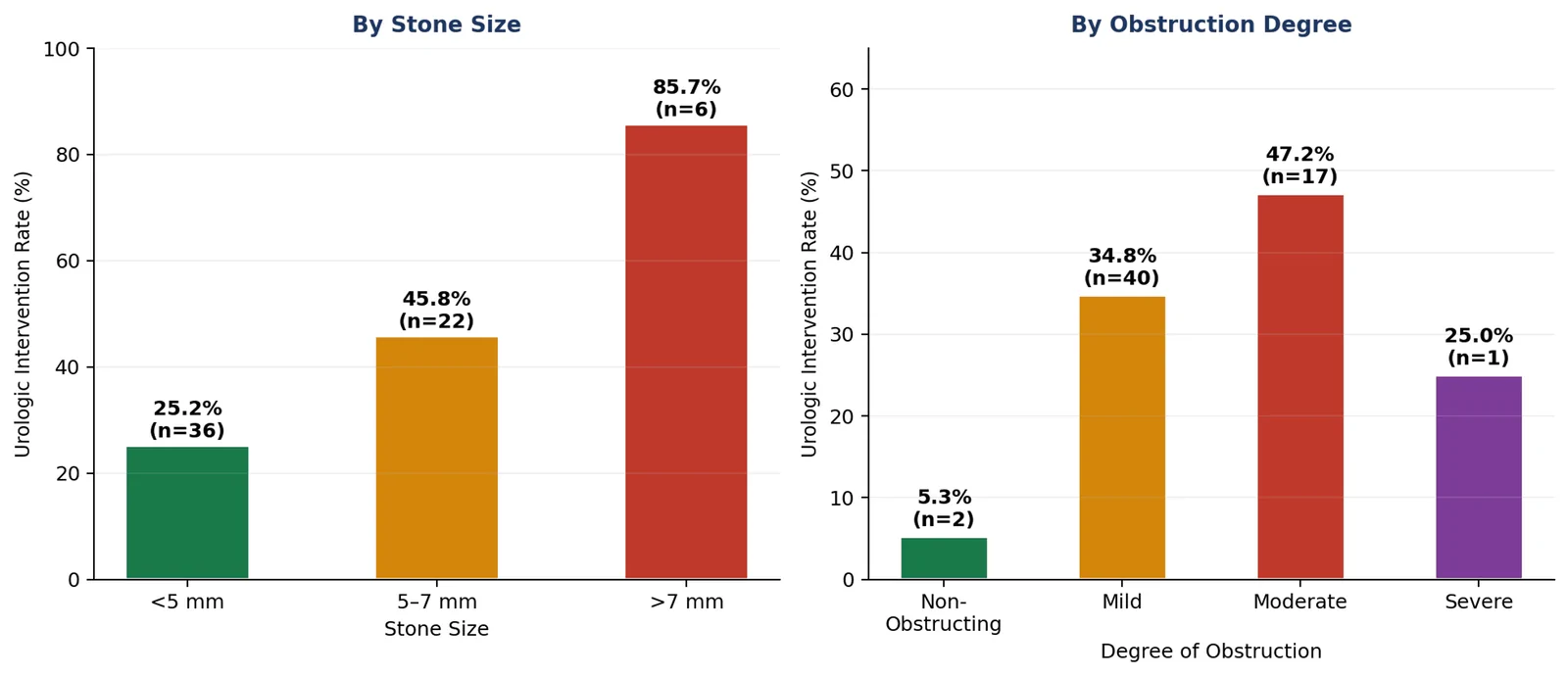
Urologic intervention rate by stone size (left) and obstruction degree (right) in the CT subgroup (n=198 with stone identified). Bars display intervention rate (%) with absolute count (n) of patients who underwent intervention.

**Figure 4:**
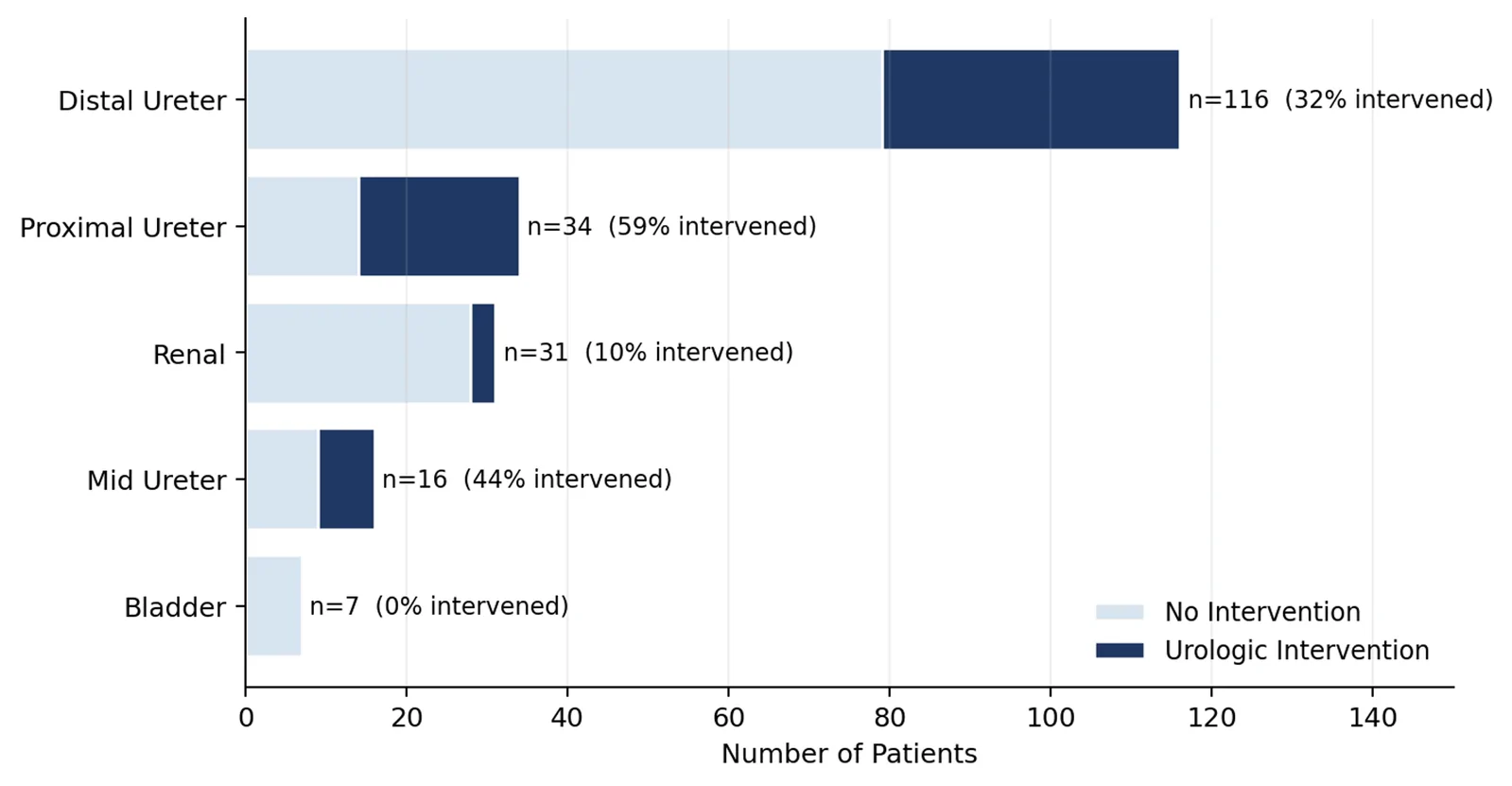
Stone location distribution and urologic intervention rates by anatomical location in the CT subgroup. Stacked bars show patients with (navy) and without (light blue) urologic intervention. The percentage of patients who underwent intervention is noted for each location.

Multivariable logistic regression: ED revisit

After adjusting for age, sex, previous stone history, opioid use, and pain severity score in a multivariable logistic regression model (n=601 with complete data; overall model: χ²=27.91, df=5, p<0.001; Nagelkerke R²=0.073), three variables remained independently associated with 14-day ED revisit (Table 4 and Figure 5): male sex (aOR 1.79; 95% CI 1.06-3.01; p=0.029), previous history of renal stones (aOR 1.88; 95% CI 1.22-2.89; p=0.004), and opioid analgesia use at the index visit (aOR 2.11; 95% CI 1.31-3.40; p=0.002). Age (aOR 0.99; p=0.229) and pain severity score (aOR 0.96; p=0.570) were not independent predictors.

**Table 4:** Multivariable Logistic Regression: Independent Predictors of ED Revisit (n=601; χ²=27.91, df=5, p<0.001; Nagelkerke R²=0.073) aOR: Adjusted odds ratio; CI: confidence interval. * p<0.05.

Variable	aOR	95% CI	p-value
Male sex (ref: Female)	1.79	1.06-3.01	0.029*
Previous history of renal stones (ref: No)	1.88	1.22-2.89	0.004*
Opioid use at index visit (ref: No)	2.11	1.31-3.40	0.002*
Age (per year increase)	0.99	0.97-1.01	0.229
Pain severity score (per unit increase)	0.96	0.83-1.11	0.570

**Figure 5:**
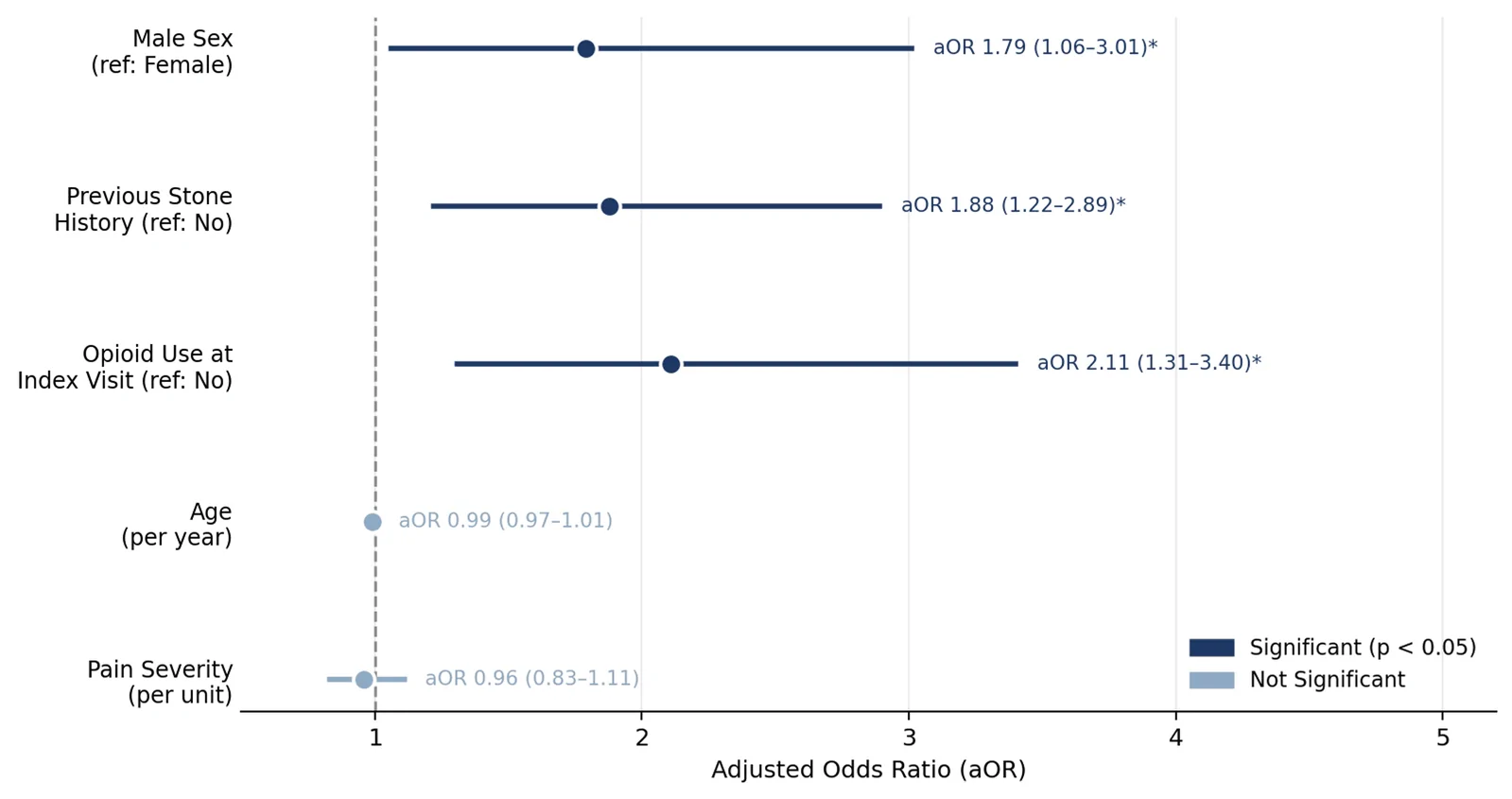
Forest plot of multivariable predictors of 14-day ED revisit (n=601). Navy points indicate statistically significant predictors (p<0.05); grey points indicate non-significant predictors. Horizontal bars represent 95% confidence intervals. Opioid use at the index visit was the strongest independent predictor (aOR 2.11; 95% CI 1.31–3.40).

Multivariable logistic regression: urologic intervention

The multivariable model for urologic intervention was restricted to the CT-imaged subgroup (n=175; overall model: χ²=34.93, df=7, p<0.001; Nagelkerke R²=0.253). Stone size <5 mm (vs 5-7 mm reference; aOR 0.43; 95% CI 0.19-0.98; p=0.044) and non-obstructing stone (vs mild obstruction; aOR 0.09; 95% CI 0.02-0.45; p=0.003) were independently protective against urologic intervention. Stone size >7 mm showed a trend toward intervention (aOR 11.26; 95% CI 0.71-178.98; p=0.086) but was limited by small numbers (n=7). Previous stone history (aOR 1.89; p=0.081) and abnormal RFT (aOR 1.82; p=0.110) trended toward significance without reaching formal thresholds. The results are presented in Table 5 and Figure 6.

**Figure 6:**
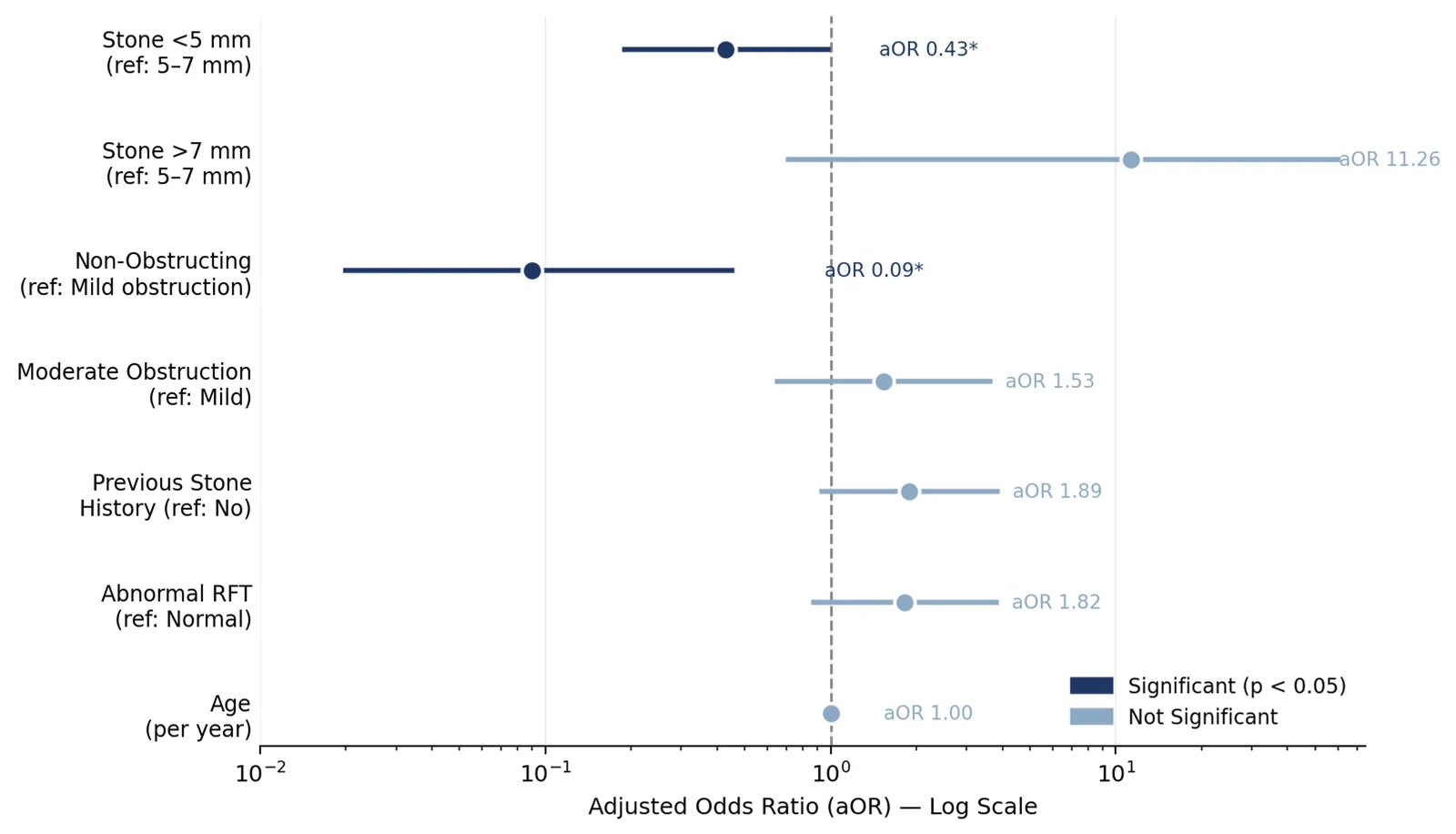
Forest plot of multivariable predictors of urologic intervention within 14 days (CT subgroup, n=175; log scale). Non-obstructing stone was the strongest protective factor against intervention (aOR 0.09; 95% CI 0.02-0.45).

**Table 5:** Multivariable Logistic Regression: Independent Predictors of Urologic Intervention (CT Subgroup, n=175; χ²=34.93, df=7, p<0.001; Nagelkerke R²=0.253) aOR = adjusted odds ratio; CI = confidence interval; RFT = renal function tests. * p < 0.05. The estimate for stone >7 mm should be interpreted cautiously given n=7 cases in this category.

Variable	aOR	95% CI	p-value
Stone size <5 mm (ref: 5-7 mm)	0.43	0.19–0.98	0.044*
Stone size >7 mm (ref: 5-7 mm)	11.26	0.71–178.98	0.086
Non-Obstructing (ref: Mild obstruction)	0.09	0.02–0.45	0.003*
Moderate obstruction (ref: Mild)	1.53	0.65–3.61	0.328
Previous history of stones (ref: No)	1.89	0.93–3.84	0.081
Abnormal RFT (ref: Normal)	1.82	0.87–3.82	0.110
Age (per year increase)	1.00	0.97–1.03	0.972

## Discussion

This retrospective cohort study of 609 patients discharged from the RMS Military Hospital ED with renal colic identified a 14-day ED revisit rate of 18.4% and a urologic intervention rate of 14.1%, consistent with, and at the lower bound, of rates reported in comparable international series [6,7]. The three independent predictors of ED revisit were male sex, previous stone history, and opioid use at the index visit. For urologic intervention, stone size and degree of obstruction were the principal imaging predictors. Pain severity score was not predictive of either outcome [9].

ED revisit rates and clinical predictors

The 14-day ED revisit rate of 18.4% is comparable to published figures from North American and European EDs [6,7]. The identification of opioid use as the strongest predictor of revisit (aOR 2.11) is clinically important. Opioid requirement may serve as a proxy marker for more severe or refractory pain rather than a direct causal factor [4]. Patients who required opioids may represent a higher-acuity subgroup with pain inadequately controlled at discharge, or may reflect prescribing patterns that predispose to early return without a robust analgesic discharge plan, supporting the use of analgesic requirement as a risk stratification variable at discharge [4,5].

Previous stone history as an independent predictor (aOR 1.88) likely reflects the natural history of recurrent urolithiasis [3]. Patients with established stone disease may have higher underlying stone burden, more complex anatomy, or established patterns of high-utilization healthcare behavior, highlighting a target population for structured outpatient stone prevention clinics [3]. The association of male sex with ED revisit (aOR 1.79) is consistent with the higher prevalence and recurrence rate of nephrolithiasis in men [2] and may inform targeted counseling strategies, although residual confounding and unmeasured behavioral factors may also contribute to this finding.

Pain severity as a non-predictor

A consistent finding was the non-significance of pain severity score as a predictor of either outcome, aligning with prior literature demonstrating that triage pain scores do not reliably correlate with stone size or downstream outcomes [9]. A composite approach incorporating analgesic requirements, stone characteristics on imaging, and patient history is more clinically informative than pain score alone [4,9].

Stone characteristics and urologic intervention

The strong protective effect of non-obstructing stones against urologic intervention (aOR 0.09) confirms the clinical principle that spontaneous passage is highly likely in the absence of significant obstruction [12,13]. Similarly, stones smaller than 5 mm were significantly less likely to require intervention than those in the 5-7 mm range (aOR 0.43), reflecting well-established spontaneous passage rates by size [12,13]. The trend toward intervention with stones >7 mm (aOR 11.26; p=0.086) was clinically plausible but underpowered given only seven such cases [8,13]. Proximal ureteral stone location was strongly associated with urologic intervention on univariate analysis (29.4% vs 10.8%), consistent with established literature on lower spontaneous passage rates of proximal stones [7,8,13]. CT imaging is the reference standard for stone characterization [10,14], though its selective use in only 36% of this cohort limits stone-based risk prediction to the CT-imaged subgroup.

Institutional context and disposition patterns

The majority of patients were referred to an outpatient urology clinic at discharge (45.9%), reflecting a conservative institutional approach with structured specialist follow-up. This safety net may partly account for the relatively low intervention and revisit rates observed [6]. Future studies could evaluate whether outpatient referral at discharge independently reduces revisit rates compared to direct discharge.

Strengths

This study has several strengths. It represents one of the largest contemporary emergency department cohorts evaluating both revisit and urologic intervention outcomes in a Middle Eastern population. The inclusion of both pre-imaging clinical variables and post-imaging stone characteristics allowed comprehensive assessment of risk stratification factors relevant to the discharge decision. Additionally, the study evaluated two clinically distinct outcomes that directly influence emergency medicine practice, providing a more complete picture of 14-day failure risk than studies examining intervention alone.

Limitations

This study has several limitations that should be considered when interpreting its findings. First, the retrospective design with reliance on electronic medical records introduces the possibility of incomplete or inconsistently documented data, though extensive standardized data cleaning was performed to mitigate this limitation. Second, CT imaging was performed in only 36% of patients at the discretion of treating physicians, limiting both the power and generalizability of stone-based predictive models; indication bias for CT may confound associations between imaging findings and outcomes. Third, the multivariable model for urologic intervention was restricted to 175 CT-imaged patients, reducing statistical power for certain variables, most notably stone size >7 mm (n=7 cases), for which the effect estimate should be interpreted cautiously. Fourth, the 14-day follow-up window was limited to the RMS Military Hospital system, and patients who sought care at other institutions would not have been captured, potentially underestimating true revisit and intervention rates. Fifth, stone passage rates and adequacy of outpatient analgesia following discharge, potentially important mediators of revisit, were not captured in the dataset. Finally, this is a single-center study from a military hospital population, which may limit generalizability to broader civilian and community emergency department settings.

## Conclusions

In this retrospective cohort of 609 patients discharged with renal colic from the RMS Military Hospital ED in Bahrain, the 14-day ED revisit rate was 18.4% and the urologic intervention rate was 14.1%. Male sex, previous stone history, and opioid analgesia requirement at the index visit were independently associated with ED revisit. Stone size ≥5-7 mm and the presence of ureteral obstruction were the principal imaging predictors of urologic intervention. Pain severity score at triage was not predictive of either outcome.

These findings provide actionable evidence to support risk stratification at discharge for renal colic patients. Clinicians should consider enhanced discharge planning, expedited outpatient follow-up, and structured analgesic optimization for patients presenting with opioid-requiring pain, recurrent stone disease, or adverse imaging characteristics. Future prospective, multi-center studies with broader CT utilization are needed to validate these findings and develop formal clinical prediction tools for this common emergency department presentation.
